# Supplementary material for: Prevalence and incidence of sexually transmitted infections among South African women initiating injectable and long-acting contraceptives
Source: PLoS One. 2023 Nov 10;18(11):e0294285. doi: 10.1371/journal.pone.0294285 (PMC10637674; doi:10.1371/journal.pone.0294285)
Supplement: S1 Questionnaire — (DOCX) [file pone.0294285.s001.docx]

Inclusivity in global research

PLOS’ policy on inclusivity in global research aims to improve transparency in the reporting of research performed outside of researchers’ own country or community and ensures that PLOS publications reporting global research adhere to high standards for research ethics and authorship. Authors of relevant research articles may be asked to complete the questionnaire below, which outlines ethical, cultural, and scientific considerations specific to inclusivity in global research. This questionnaire may be requested when researchers have travelled to a different country to conduct research, if research uses samples collected in another country, research with Indigenous populations or their lands, or if research is on cultural artefacts. Researchers travelling to another country solely to use laboratory equipment will not normally be required to complete the questionnaire. However, the questionnaire can be requested at the journal’s discretion for any submission – if you have been requested to complete this questionnaire by the PLOS journal you submitted to, please do so.

Please complete the questionnaire below and include this as a Supporting Information file with your manuscript. Note that if your paper is accepted for publication, this checklist will be published with your article in the supporting information files. Please ensure that you reference the checklist in the main body of your manuscript. We suggest adding a subsection ‘Inclusivity in global research’ to your Methods section and adding the following sentence: “Additional information regarding the ethical, cultural, and scientific considerations specific to inclusivity in global research is included in the Supporting Information (SX Checklist)”

The questions have been designed to be applicable to a wide range of study types, and there are subsections for both human subjects research and non-human subjects research. If any of the questions are not relevant to your research please mark them as “N/A” as appropriate.

**Ethical considerations, permits and authorship**

*This section is applicable to all research types.*

Provide details as to who granted permissions and/or consent for the study to take place in the Methods section of your manuscript. This should include the names of **all** ethics boards, governmental organizations, community leaders or other bodies that provided approval for the study. If individuals provided approval refer to these people by their role or title but do not list their name(s).

Reported on page number: 5

If there were any deviations from the study protocol after approval was obtained please provide details of these changes in the Methods section of your manuscript.
Did this study involve local collaborators that are residents of the country where the research was conducted or members of the community studied? If you do not have any authors from said communities, please provide an explanation for this below.

There were no protocol deviations.

Yes, Rushil Harryparsad, Bahiah Meyer, Ongeziwe Taku, Anna-Lise Williamson, Jennifer Smit, Khatija Ahmed, Mags Beksinska were residents of South Africa throughout the study. Celia Mehou-Loko and Lindi Masson were residents of South Africa for the majority of the study.

Everyone listed as an author should meet PLOS’ criteria for authorship and all individuals who meet these criteria should be included in the author byline, rather than the acknowledgements. For further information please see the journal’s Authorship Policy.

**Human subjects research (e.g. health research, medical research, cross-cultural psychology)**

Did you obtain written informed consent from a representative of the local community or region before the research took place? How did you establish who speaks for the community? Details of written informed consent obtained from study participants should be reported separately in the Methods section of your manuscript.

The study was presented to the Community Advisory Boards (CABs) at the two study sites in advance of the study and the CABs provided input, however written informed consent was not obtained from community representatives.

How did members of the local community provide input on the aims of the research investigation, its methodology, and its anticipated outcome(s)?

The Community Advisory Boards (CAB), who are volunteers and serve in an advisory capacity to the sites provide input on important aspects of the trials conducted. They represent key populations and sectors within the community and were consulted and given the opportunity to provide inputs on the feasibility, social and cultural acceptability of the methodology and design of the research, as well as determine if the research outcomes would be of value to the community. These CAB members have also been trained on research literacy and HIV-related issues, clinical trial methods and procedures, research ethics, and the principles of Good Participatory Practice. These CABs serves as a representation of the voices of the community .

Across all study sites for the ECHO parent trial there was a Global Community Advisory Group (GCAG). The ECHO GCAG included a subset of advocates from the The Hormonal contraception-HIV Advocacy Working Group which includes highly visible women leaders from diverse geographies who advocate on the global, national and local stage. The GCAG advised and communicated regularly with the ECHO Consortium around key trial issues-particularly community engagement.

When engaging with the local community, how did you ensure that the informed consent documents and other materials could be understood by local stakeholders?

SRC and Wits MRU had created a community engagement plan that included ongoing and planned initiatives engaging various community stakeholders.

The Community Liaison Officers (CLO) and Recruiting Officers educated and recruited the community by following the GPP principles. The community team made certain that study-related documentation, such as informed consent and educational materials (approved by ethics), were distributed in the language that stakeholders were most comfortable with. In addition, the materials were presented utilizing platforms that were most appropriate for the intended community, giving them the opportunity to ask questions and seek clarification. We also utilised tools to ascertain participant understanding of the consent such as "informed consent comprehension assessment".

Will the findings of the research be made available in an understandable format to stakeholders in the community where the study was conducted (e.g. via a presentation, summary report, copies of publications, etc.)? Please provide details of how this will be achieved.

SRC: CAB members will be invited and told about the results immediately following the staff meeting at which the study's findings will be shared. Stakeholders such as Public Health Officials and the scientific community will be presented with the results during meetings. For the larger community, presentations will be held during town hall meetings to address the community.

Wits MRU site: As results become available the adult and adolescent CABs at Wits MRU will be informed at the next standing 3-monthly meeting. Results will also be made available to various stakeholders in the province (Departments of Health, Non-governmental organisations etc) via the Wits MRU Dissemination days and other research days held locally.

**Non-human subjects research using specimens/ animals collected as part of the study, or those housed in archival collections. Examples include archaeology, paleontology, botany and zoology.**

Did the permission you obtained from a local authority to perform the study include an agreement on access to outputs and benefit sharing? This may include procedures to enable fair distribution of the benefits and resources arising from the research performed. Please include any details of Prior Informed Consent and Benefit Sharing Agreements obtained. These may be required by field-specific regulations, for example the Convention on Biological Diversity (CBD) and the associated Nagoya Protocol.

If the material used in your study was imported, please A) provide the year it was imported and B) indicate whether permits were obtained to import/export the materials used, C) provide details of any permits obtained. If this information is not available, please indicate this.

If you used archival specimens, please state how the material used in your study was acquired by the institute it is held in and provide details of any permits obtained for the original excavations/ sample collection. If this information is not available, please indicate this.

How was the potential cultural significance of the materials collected in your study to local communities considered in your research design? Were Indigenous peoples and/or local researchers and institutions involved with archaeological excavations / collection of specimens? If so, please provide a description of their involvement.

If your manuscript includes photographs of human remains please indicate whether authors obtained permission from descendants or affiliated cultural communities to do so.
