# Supplementary figures and images for: Prevalence and incidence of sexually transmitted infections among South African women initiating injectable and long-acting contraceptives
Source: PLoS One. 2023 Nov 10;18(11):e0294285. doi: 10.1371/journal.pone.0294285 (PMC10637674; doi:10.1371/journal.pone.0294285)

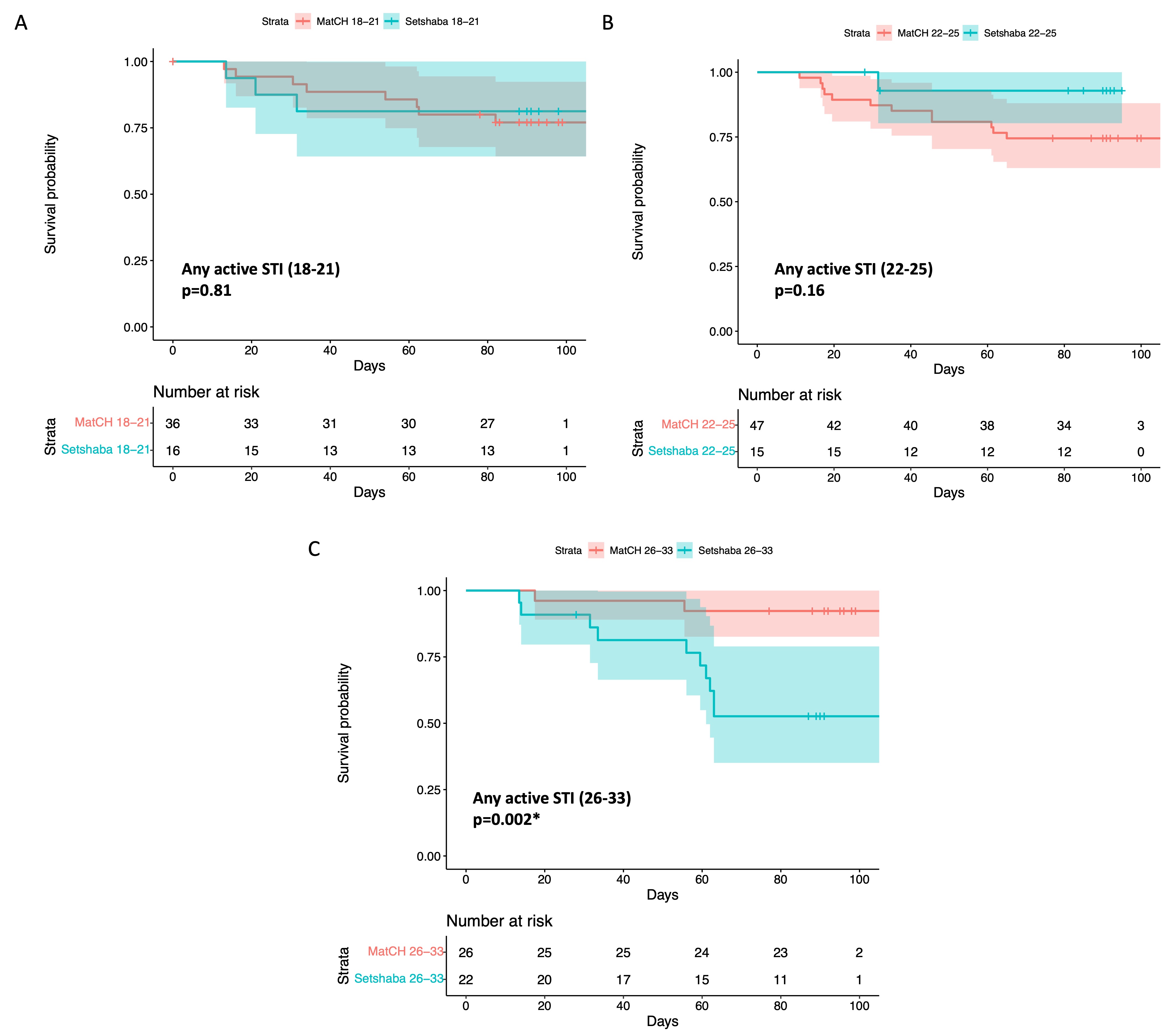

Supplement: S2 Fig — (A-C). Kaplan-Meier curve showing sexually transmitted infection (STI) incidence between study site by age group over time (days). Red represents the MatCH site, green the Setshaba site. The tables show the number of individuals at risk of acquiring STIs over time. A vertical drop represents an event has occurred and a verticle tick mark on the curves indicate that a participant has been censored at that timepoint. (A), STI incidence between sites for 18–21 year olds were not statistically significant. (B), There was no statistically significant differences for 25–33 years olds between sites. (C), STI incidence between sites for 26–33 year olds had a statistically significant difference (p = 0.002). (JPG) [file pone.0294285.s003.jpg]

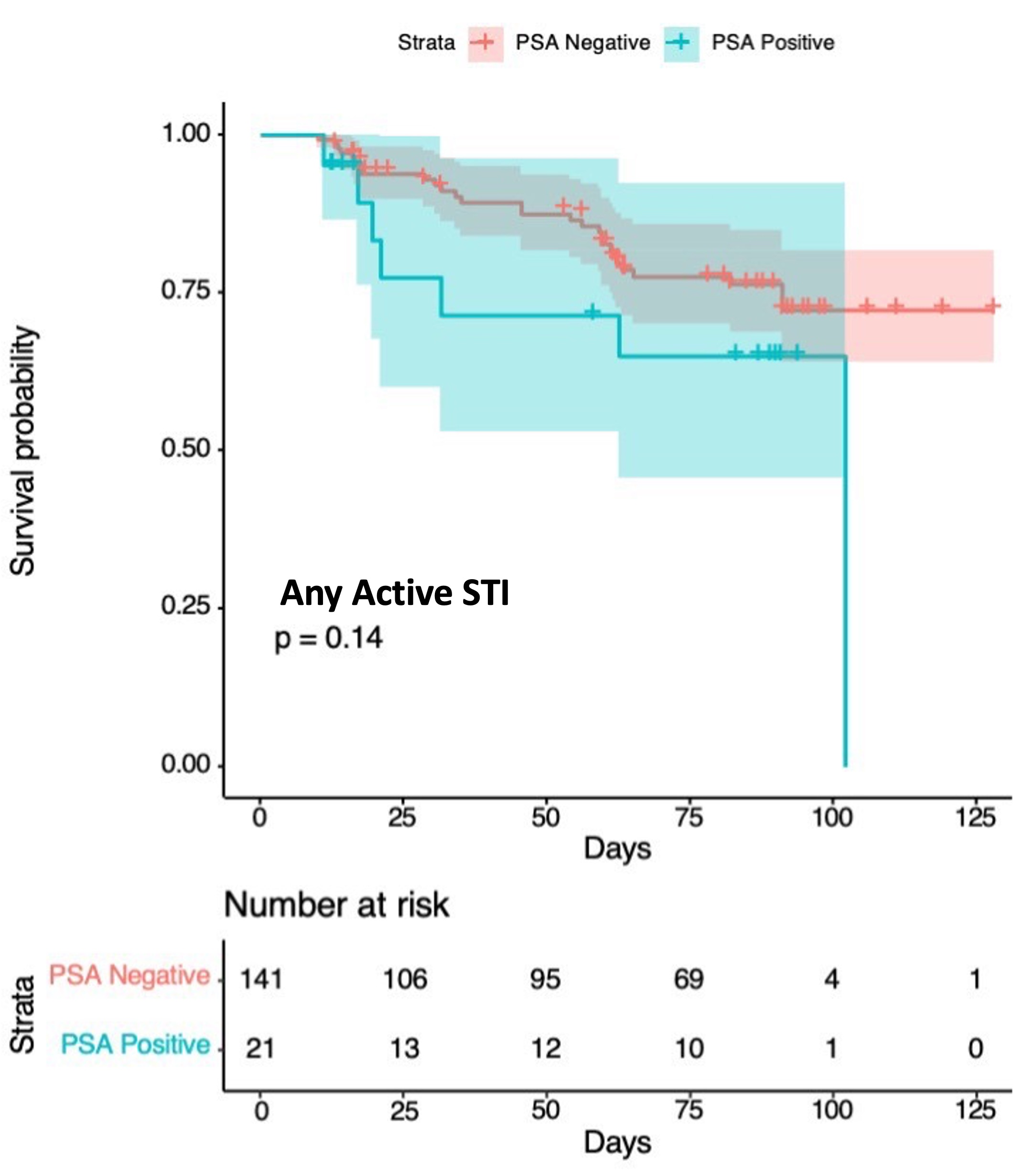

Supplement: S3 Fig — Red represents participants positive for PSA at any visit, green indicates participants negative for PSA at all visits. The tables show the number of individuals at risk of acquiring any active STI over time. A vertical drop represents an event has occurred and a cross mark on the curves indicates that a participant has been censored at that timepoint. There was no statistically significant difference in the incidence of an active STI between participants positive or negative for PSA. (JPG) [file pone.0294285.s004.jpg]

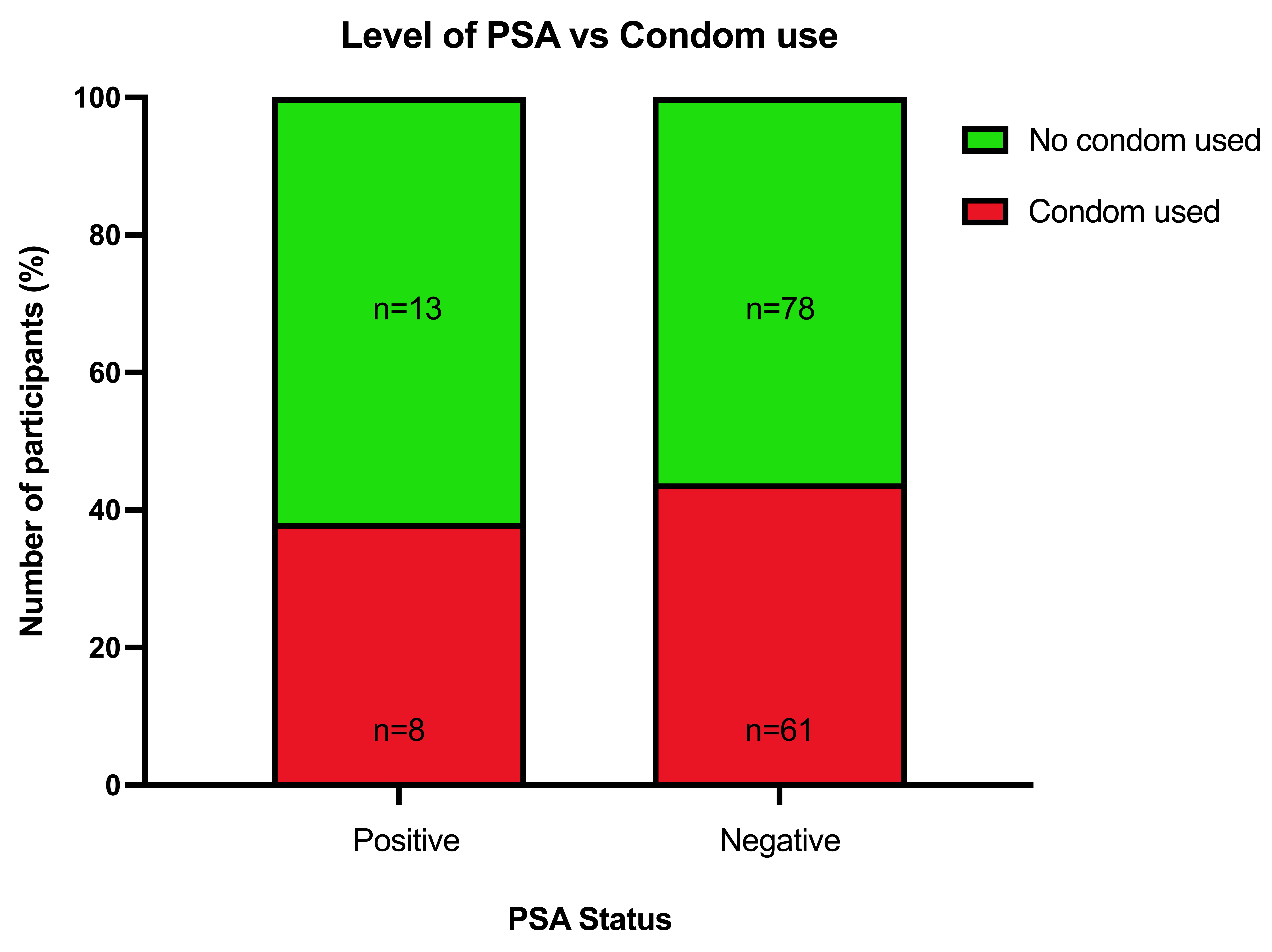

Supplement: S4 Fig — There were no significant differences in PSA detection in women who reported condom use and in women reporting no condom usage. (JPG) [file pone.0294285.s005.jpg]

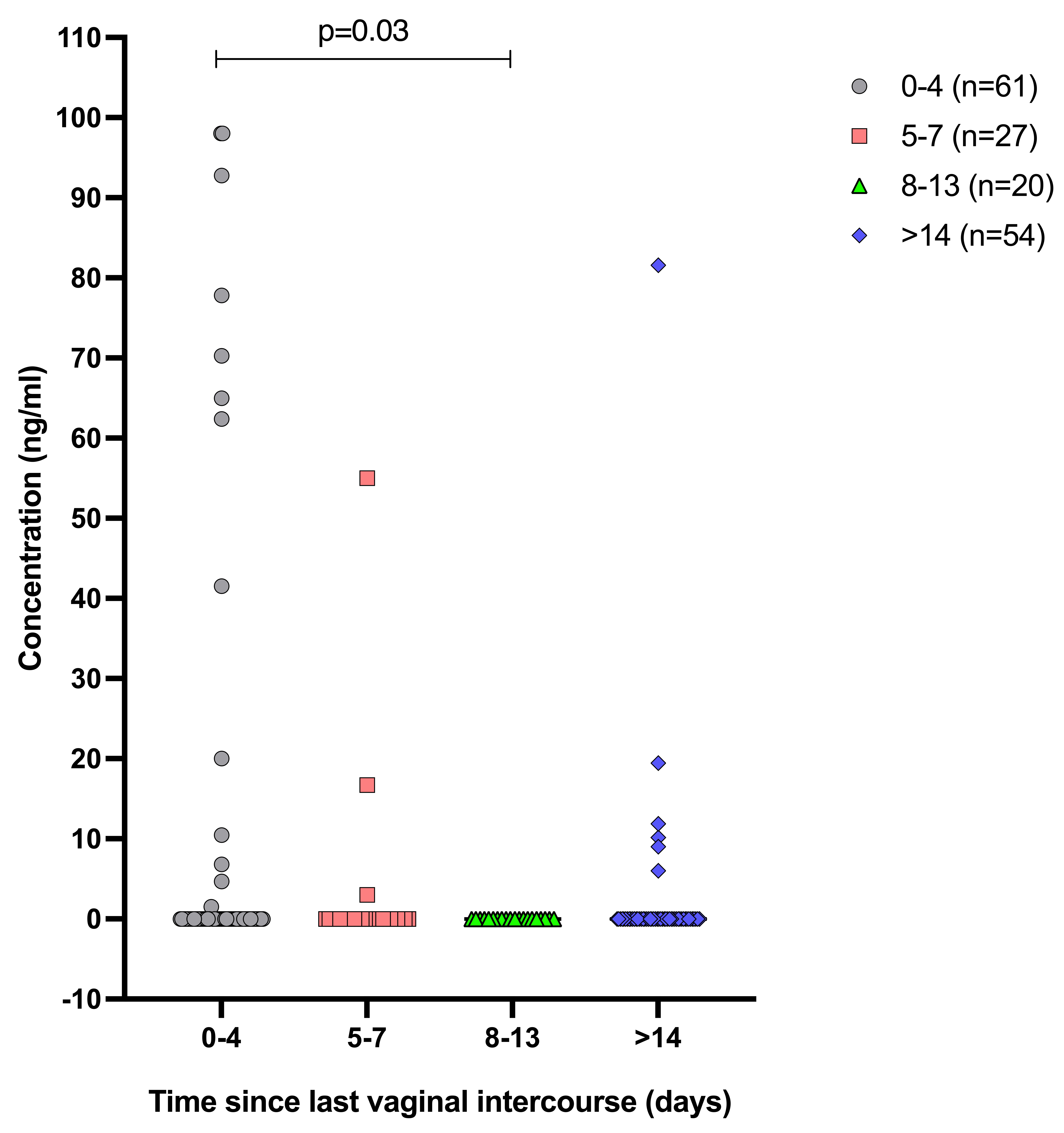

Supplement: S5 Fig — PSA concentration was significantly higher among women reporting vaginal intercourse 0–4 days compared to 8–13 days prior to the study visit (p = 0.03). (JPG) [file pone.0294285.s006.jpg]
