# Supplementary material for: Prevalence and incidence of sexually transmitted infections among South African women initiating injectable and long-acting contraceptives
Source: PLoS One. 2023 Nov 10;18(11):e0294285. doi: 10.1371/journal.pone.0294285 (PMC10637674; doi:10.1371/journal.pone.0294285)
Supplement: S1 Table — Abbreviations: ¥ n = 107;kg, kilogram; cm, centimeter. Proportions were compared using the Chi-Square test and continuous variables were compared using Kruskal-Wallis test. *p<0.05 following Bonferroni correction was considered statistically significant. (DOCX) [file pone.0294285.s007.docx]

| ​ | | **Total**  **(n=162)** | | **MatCH**  **(n=109)** | | **Setshaba**  **(n=53)** | | **p-value** | |
| --- | --- | --- | --- | --- | --- | --- | --- | --- | --- |
| Age  [mean(range)] | | 24 (18-33) | | 23.5 (18-33) | | 24.6 (18-33) | | 0.16 | |
| Education [n(%)]​ | |  | |  | |  | | **<0.01*** | |
| Secondary​ | | 126 (78) | | 78 (71.6) | | 48 (90.6) | |  | |
| Post-secondary ​ | | 36 (22) | | 31 (28.4) | | 5 (9.4) | |  | |
| Weight (kg)  [mean(range)]​ | | 68.9  (40.7-135.2) | | 67.5  (40.7-107) | | 71.8  (44.2-135.2) | | 0.33 | |
| Height (cm)  [mean(range)]​ | | 158.6  (143-188) | | 158.1  (143-172) | | 159.6  (143-188) | | 0.35 | |
| Body mass index  [mean(range)]​ | | 27.4  (17.2-52.2) | | 27.0  (17.2-41.9) | | 28.2  (17.9-52.2) | | 0.58 | |
| Vaginal intercourse in past 3 months [mean(range)]​ | | 16  (0-60) | | 11  (0-48) | | 27  (1-60) | | **<0.0001*** | |
| Marital status [n(%)]​ | |  | |  | |  | | >0.99 | |
| Married​ | | 1 (1) | | 1 (0.9) | | 0 (0) | |  | |
| Not Married​ | | 161 (99.4) | | 108 (99.1) | | 53 (100) | |  | |
| Current Smoker [n(%)]​ | | 37 (23) | | 7 (6.4) | | 30 (56.6) | | **<0.0001*** | |
| Prostate specific antigen  detected [n(%)]​ | | 21 (13) | | 13 (11.9) | | 8 (15.1) | | 0.62 | |
| Ever use condoms [n(%)]​ | |  | |  | |  | | 0.39 | |
| Yes​ | | 69 (43) | | 49 (45.8)^¥^ | | 20 (37.7) | |  | |
| No​ | | 91 (56) | | 58 (54.2)^¥^ | | 33 (62.3) | |  | |
| Abbreviations: ^¥^ n=107;kg, kilogram; cm, centimeter. Proportions were compared using the Chi-Square test and continuous variables were compared using Kruskal-Wallis test. *p<0.05 following Bonferroni correction was considered statistically significant. | |  | | ​ | | ​ | |  | |

**Table S1:** **Baseline demographic, behavioural and clinical characteristics of study by study site**
