# Supplementary material for: Prevalence and incidence of sexually transmitted infections among South African women initiating injectable and long-acting contraceptives
Source: PLoS One. 2023 Nov 10;18(11):e0294285. doi: 10.1371/journal.pone.0294285 (PMC10637674; doi:10.1371/journal.pone.0294285)
Supplement: S2 Table — Abbreviations: STI, sexually transmitted infection. ^tested positive for at least one STI (CT, NG, TV, MG or HSV-2). Proportions were compared using the Fishers Exact test *p<0.05 following Bonferroni correction was considered statistically significant. (DOCX) [file pone.0294285.s008.docx]

|  |  | Study Site n (%) | |  |
| --- | --- | --- | --- | --- |
| STI | Total  (n=162) | MatCH  (n=109) | Setshaba  (n=53) | p-value |
| Any active STI​^ | 64 (40)​ | 39 (36)​ | 25 (47)​ | 0.17​ |
| *Chlamydia*  *trachomatis*​ | 31 (19)​ | 21 (19)​ | 10 (19)​ | >0.99​ |
| *Neisseria*  *gonorrhoeae*​ | 7 (4) ​ | 3 (3)​ | 4 (8)​ | 0.22​ |
| Herpes simplex  virus 2 shedding​ | 9 (6)​ | 8 (7)​ | 1 (2)​ | 0.27​ |
| *Trichomonas*  *vaginalis*​ | 31 (19)​ | 14 (13)​ | 17 (32)​ | **<0.01**​* |
| *Mycoplasma*  *genitalium*​ | 7 (4) ​ | 4 (4)​ | 3 (6)​ | 0.68​ |
| Multiple active STIs | 16 (10) | 9 (8) | 7 (13) | 0.40 |

**Table S2: Baseline STI prevalence overall and by study site**

Abbreviations: STI, sexually transmitted infection. ^tested positive for at least one STI (CT, NG, TV, MG or HSV-2). Proportions were compared using the Fishers Exact test *p<0.05 following Bonferroni correction was considered statistically significant.
