# Supplementary material for: Prevalence and incidence of sexually transmitted infections among South African women initiating injectable and long-acting contraceptives
Source: PLoS One. 2023 Nov 10;18(11):e0294285. doi: 10.1371/journal.pone.0294285 (PMC10637674; doi:10.1371/journal.pone.0294285)
Supplement: S3 Table — Abbreviations: STI, sexually transmitted infection. ^tested positive for at least one STI (CT, NG, TV, MG or HSV-2). Proportions were compared using the Fishers Exact test *p<0.05 following Bonferroni correction was considered statistically significant. (DOCX) [file pone.0294285.s009.docx]

|  |  | **Contraceptive group n (%)** | | | **p-value** | | |
| --- | --- | --- | --- | --- | --- | --- | --- |
| STI | **Total**  **(n=162)**  **n (%)** | **LNG implant**  **(n=58)** | **DMPA-IM**  **(n=53)** | **Cu-IUD**  **(n=51)** | **LNG**  **implant vs DMPA-IM** | **LNG**  **implant vs Cu-IUD** | **DMPA-IM**  **vs Cu-IUD** |
| Any active STI^ | 64 (40) | 29 (50) | 18 (34) | 17 (33) | 0.12 | 0.09 | >0.99 |
| *Chlamydia*  *trachomatis* | 31 (19) | 16 (28) | 8 (15) | 7 (14) | 0.10 | 0.10 | >0.99 |
| *Neisseria*  *gonorrhoeae* | 7 (4) | 3 (5) | 2 (4) | 2 (4) | >0.99 | >0.99 | >0.99 |
| *Trichomonas*  *vaginalis* | 31 (19) | 12 (21) | 9 (17) | 10 (20) | 0.23 | 0.47 | >0.99 |
| *Mycoplasma*  *genitalium* | 7 (4) | 4 (7) | 2 (4) | 1 (2) | 0.62 | 0.62 | >0.99 |
| Herpes simplex  virus 2 shedding | 9 (6) | 2 (3) | 4 (8) | 3 (6) | 0.31 | >0.99 | 0.29 |
| Herpes simplex  virus 2 serology | 54 (38) | 18 (35) | 21 (46) | 15 (33) | 0.23 | 0.47 | >0.99 |
| Multiple active STIs | 16 (10) | 6 (10) | 6 (11) | 4 (8) | >0.99 | 0.75 | 0.74 |

**Table S3:** **Baseline STI prevalence overall and by contraceptive group**

Abbreviations: STI, sexually transmitted infection. ^tested positive for at least one STI (CT, NG, TV, MG or HSV-2). Proportions were compared using the Fishers Exact test *p<0.05 following Bonferroni correction was considered statistically significant.
