# Supplementary material for: Prevalence and incidence of sexually transmitted infections among South African women initiating injectable and long-acting contraceptives
Source: PLoS One. 2023 Nov 10;18(11):e0294285. doi: 10.1371/journal.pone.0294285 (PMC10637674; doi:10.1371/journal.pone.0294285)
Supplement: S4 Table — *p<0.05 following Bonferroni correction was considered statistically significant. (DOCX) [file pone.0294285.s010.docx]

|  |  | Study Site | |  |
| --- | --- | --- | --- | --- |
| STI | **Total**  **(n/100wy)** | **MatCH**​  **(n/100wy)**​ | **Setshaba**  **(n/100wy)**​ | **p-value**​ |
| Any active STI​ | 107.93​ | 95.1​ | 137.0 | 0.33​ |
| *Chlamydia trachomatis*​ | 40.39​ | 39.0 | 43.1​ | 0.88​ |
| *Neisseria gonorrhoeae*​ | 7.66​ | 3.7​ | 16.3​ | 0.22​ |
| *Trichomonas vaginalis*​ | 43.66​ | 38.9 | 58.1​ | 0.49​ |
| *Mycoplasma genitalium*​ | 18.02​ | 19.1​ | 15.8​ | 0.85​ |
| Multiple active STIs | 35.51 | 48.9 | 8.3 | 0.06 |

**Table S4: Three-month STI incidence overall and by study site**

*p<0.05 following Bonferroni correction was considered statistically significant.
