# Supplementary material for: Prevalence and incidence of sexually transmitted infections among South African women initiating injectable and long-acting contraceptives
Source: PLoS One. 2023 Nov 10;18(11):e0294285. doi: 10.1371/journal.pone.0294285 (PMC10637674; doi:10.1371/journal.pone.0294285)
Supplement: Attachment — Submitted filename: Harryparsad et al. Reviewer Response_3 May 2023.pdf [file pone.0294285.s012.pdf]

Burnet Institute
Medical Research. Practical Action.

DIRECTOR and CEO – Professor Brendan Crabb AC PhD
CHIEF PATRON – The Honourable Linda Dessau AC, Governor of Victoria

3rd May 2023

Dear Editor,

RE: Response to the Reviewers

Reviewer comment:
Based on our evaluation of your manuscript and the information you provided, we do not feel that your submission meets our ethical requirements for human subjects research submissions. PLOS ONE requires that research meets all applicable standards for the ethics of experimentation and research integrity (http://journals.plos.org/plosone/s/human-subjects-research). We reserve the right to reject any submission that does not meet our internal ethical standards, which in some cases are more stringent than local ethical standards.

We understand that you obtained ethical approval from your local ethics committee after you started recruiting participants. PLOS ONE does not accept retrospective ethical approval for human subjects research. As a result, we cannot consider the manuscript for publication at PLOS ONE.

Response:
We apologize that our ethical approval and enrolment dates were not clear in our original submission documents. The dates listed in the manuscript were the dates for the entire parent study of 7800 women, however our sub-cohort of 162 women was enrolled towards the end of the parent study, only after ethical approval was obtained to store biological specimens for future research at both clinical sites (dated 25 April 2017 and 19 May 2017). After our funding for the laboratory research was received, we applied for and received University of Cape Town ethical approval (dated 27 November 2018) and we submitted and received approval for a further amendment through the University of the Witwatersrand to detail the biological studies that would be conducted (dated 22 January 2019). We have edited the manuscript to clarify the dates of enrolment and provided additional verification of ethical approval.

Methods, Page 5:

Study participants
The participants in this sub-study were enrolled and completed follow-up at the Setshaba Research Centre (SRC) in Tshwane (n=53) and MatCH Research Unit (MRU) in eThekwini (n=109) between June 2017 and February 2018 as part of the ECHO trial (18). ECHO evaluated HIV incidence among 7800 women randomised to DMPA-IM, the LNG implant or the copper-IUD in South Africa, Kenya, Zambia and Eswatini ~~between December 2015 and September 2017~~ (18).

Thank you for considering our revised submission.

Yours sincerely,

Masson.

Dr Lindi Masson

Senior Research Fellow
Life Sciences Discipline
Burnet Institute
Australia

Honorary Research Associate
Department of Pathology
University of Cape Town
South Africa

Honorary Scientist
Centre for the AIDS Programme of Research in South Africa
South Africa

Adjunct Senior Lecturer
Monash University
Australia

Head Office
85 Commercial Road, Melbourne, Victoria, Australia 3004
GPO Box 2284, Melbourne, Victoria, Australia 3001
Tel +61 3 9282 2111 **Fax** +61 3 9282 2100 **Email** info@burnet.edu.au **burnet.edu.au**

Macfarlane Burnet Institute for Medical Research and Public Health Ltd. ABN 49 007 349 984
