# Supplementary material for: Prevalence and incidence of sexually transmitted infections among South African women initiating injectable and long-acting contraceptives
Source: PLoS One. 2023 Nov 10;18(11):e0294285. doi: 10.1371/journal.pone.0294285 (PMC10637674; doi:10.1371/journal.pone.0294285)
Supplement: Attachment — Submitted filename: STI paper response to editor comments_27 Sep 2023.docx [file pone.0294285.s013.docx]

**Harryparsad *et al*. Manuscript Response to Editor Comments**

**PONE-D-23-13490R1**

**a)        Please clarify the sources of funding (financial or material support) for your study. List the grants or organizations that supported your study, including funding received from your institution.**

Response: The research reported in this publication was supported by the Eunice Kennedy Shriver National Institute of Child Health & Human Development of the National Institute of Health under Award Number R01HD096937-01 (PIs: Morrison, Deese and Masson). The content is solely the responsibility of the authors and does not necessarily represent the official views of the National Institutes of Health. LM was supported by the Carnegie Corporation of New York, South African National Research Foundation and Australian National Health and Medical Research Council. The Evidence for Contraceptive Options and HIV Outcomes (ECHO) Trial was supported by the combined generous support of the Bill & Melinda Gates Foundation [grant OPP1032115]; the American people through the United States Agency for International Development [grant AID-OAA-A-15-00045] the Swedish International Development Cooperation Agency [grant 2017/762965-0]; the South Africa Medical Research Council; and the United Nations Population Fund. Contraceptive supplies were donated by the Government of South Africa and US Agency for International Development.

**b)        State what role the funders took in the study. If the funders had no role in your study, please state: “The funders had no role in study design, data collection and analysis, decision to publish, or preparation of the manuscript.”**

Response: The funders had no role in study design, data collection and analysis, decision to publish, or preparation of the manuscript

**c)        If any authors received a salary from any of your funders, please state which authors and which funders.**

Response: LM, MS, PLC, XG, CML, FLDH, JS, JS, KN, KH, MB, GB, CM and JD received salary from the National Institute of Health.

RH, BM, OT received student stipends from the National Institute of Health.

LM also received salary from the Carnegie Corporation of New York, South African National Research Foundation and Australian National Health and Medical Research Council.

**d)        If you did not receive any funding for this study, please state: “The authors received no specific funding for this work.”**

Response: N/A

**2.  We note that you have included the phrase “data not shown” in your manuscript. Unfortunately, this does not meet our data sharing requirements. PLOS does not permit references to inaccessible data. We require that authors provide all relevant data within the paper, Supporting Information files, or in an acceptable, public repository. Please add a citation to support this phrase or upload the data that corresponds with these findings to a stable repository (such as Figshare or Dryad) and provide and URLs, DOIs, or accession numbers that may be used to access these data. Or, if the data are not a core part of the research being presented in your study, we ask that you remove the phrase that refers to these data.**

Response: We apologise for this and have now provided the data as Figures S3, S4 and S5.

**3. Please include a complete copy of PLOS’ questionnaire on inclusivity in global research in your revised manuscript. Our policy for research in this area aims to improve transparency in the reporting of research performed outside of researchers’ own country or community. The policy applies to researchers who have travelled to a different country to conduct research, research with Indigenous populations or their lands, and research on cultural artefacts. The questionnaire can also be requested at the journal’s discretion for any other submissions, even if these conditions are not met. Please find more information on the policy and a link to download a blank copy of the questionnaire here:** [**https://aus01.safelinks.protection.outlook.com/?url=https%3A%2F%2Fjournals.plos.org%2Fplosone%2Fs%2Fbest-practices-in-research-reporting&data=05%7C01%7CLindi.Masson%40burnet.edu.au%7C44baf172456a4dc60d1108dbb0d70be1%7Cc471a057a4e746c596342e95c39663fa%7C0%7C0%7C638298209897733676%7CUnknown%7CTWFpbGZsb3d8eyJWIjoiMC4wLjAwMDAiLCJQIjoiV2luMzIiLCJBTiI6Ik1haWwiLCJXVCI6Mn0%3D%7C3000%7C%7C%7C&sdata=eAoTSX40H8mf6ERJt3Uyp11YS6ZXEpY7Mt8dfIhi%2FbM%3D&reserved=0**](https://aus01.safelinks.protection.outlook.com/?url=https%3A%2F%2Fjournals.plos.org%2Fplosone%2Fs%2Fbest-practices-in-research-reporting&data=05%7C01%7CLindi.Masson%40burnet.edu.au%7C6ef3cef3855e4847034608dbb4889b2a%7Cc471a057a4e746c596342e95c39663fa%7C0%7C0%7C638302271095991907%7CUnknown%7CTWFpbGZsb3d8eyJWIjoiMC4wLjAwMDAiLCJQIjoiV2luMzIiLCJBTiI6Ik1haWwiLCJXVCI6Mn0%3D%7C3000%7C%7C%7C&sdata=pOLXNIZADWLCnfgG8CbgS6wYozdTVG6PQeneQnsfXCo%3D&reserved=0)**. Please upload a completed version of your questionnaire as Supporting Information when you resubmit your manuscript.**

Response: We have uploaded this questionnaire as requested.

**4. I note that in your Data Availability statement you state: “The data that support the findings of this study are available from the corresponding author upon reasonable request."

Please note that in the interest of long-term data availability, PLOS data policy states that it is not acceptable for an author to be the sole named individual responsible for ensuring data access (**[**https://aus01.safelinks.protection.outlook.com/?url=https%3A%2F%2Fjournals.plos.org%2Fplosone%2Fs%2Fdata-availability%23loc-acceptable-data-access-restrictions&data=05%7C01%7CLindi.Masson%40burnet.edu.au%7C44baf172456a4dc60d1108dbb0d70be1%7Cc471a057a4e746c596342e95c39663fa%7C0%7C0%7C638298209897733676%7CUnknown%7CTWFpbGZsb3d8eyJWIjoiMC4wLjAwMDAiLCJQIjoiV2luMzIiLCJBTiI6Ik1haWwiLCJXVCI6Mn0%3D%7C3000%7C%7C%7C&sdata=FMXOvN2Mannty31us3pGQc7SwKPnBQjzI9h9%2B7e2iK0%3D&reserved=0**](https://aus01.safelinks.protection.outlook.com/?url=https%3A%2F%2Fjournals.plos.org%2Fplosone%2Fs%2Fdata-availability%23loc-acceptable-data-access-restrictions&data=05%7C01%7CLindi.Masson%40burnet.edu.au%7C6ef3cef3855e4847034608dbb4889b2a%7Cc471a057a4e746c596342e95c39663fa%7C0%7C0%7C638302271095991907%7CUnknown%7CTWFpbGZsb3d8eyJWIjoiMC4wLjAwMDAiLCJQIjoiV2luMzIiLCJBTiI6Ik1haWwiLCJXVCI6Mn0%3D%7C3000%7C%7C%7C&sdata=91v2CWxlXECB6gWdT1odAUW%2FOOBssfn%2FuNYg1ET4zPU%3D&reserved=0)**).

You can, however, still have the data available upon request if you provide a non-author contact (e.g., a Research Ethics Committee or Institutional Review Board contact). You can update your Data Availability statement in your cover letter when you resubmit your revised manuscript, as we will not be able to do it whilst the manuscript is still with you or in your account.**

Response: We have now included all data as Table S5.
